# TNM Staging of Colorectal Cancer Should be Reconsidered According to Weighting of the T Stage: Verification Based on a 25-Year Follow-Up

**DOI:** 10.1097/MD.0000000000002711

**Published:** 2016-02-12

**Authors:** Jun Li, Cheng-Hao Yi, Ye-Ting Hu, Jin-Song Li, Ying Yuan, Su-Zhan Zhang, Shu Zheng, Ke-Feng Ding

**Affiliations:** From the Department of Surgical Oncology (JL, S-ZZ, K-FD), Second Affiliated Hospital, Zhejiang University School of Medicine; Zhejiang University Cancer Institute and the Key Laboratory of Cancer Prevention and Intervention (JL, C-HY, Y-TH, YY, S-ZZ, SZ, K-FD), China National Ministry of Education; EMR and Intelligent Expert System Engineering Research Center (J-SL), the Key Laboratory of Biomedical Engineering, China National Ministry of Education, Zhejiang University College of Biomedical Engineering and Instrument Science; and Department of Medical Oncology (YY), Second Affiliated Hospital, Zhejiang University School of Medicine, Hangzhou, Zhejiang Province, China.

## Abstract

The gradient monotonicity of existing tumor, node, metastases staging systems for colorectal cancer is unsatisfactory. Our proposed T-plus staging system strengthens weighting of the T stage. In this study, applicability of the T-plus staging system was verified with data of a Chinese colorectal cancer center.

Records of 2080 nonmetastatic, advanced cancer patients undergoing colorectal cancer surgery from 1985 to 2011 were reviewed for T, N stage pathology and follow-up information. Using overall and disease-specific survival data, the 7th edition tumor, node, metastases staging system and the T-plus staging system were compared for stage homogeneity and discrimination and gradient monotonicity.

For gradient monotonicity, the T-plus staging system was superior for both colon and rectal cancer. With Kaplan–Meier survival curves, the T-plus staging system discriminated among different stages, and the corresponding survival was inversely associated with the stage. However, for the 7th edition tumor, node, metastases staging system, stage IIIa had a better prognosis than stage II for rectal cancer and stage I for colon cancer. For homogeneity within the same stage and discrimination between different stages, the 2 staging systems were similar for colorectal cancer, but the T-plus system was clearly better for colon cancer.

The T-plus staging system provides good gradient monotonicity. For future colorectal cancer staging systems, we propose replacement of lymph node status as the criterion to discriminate colorectal cancer stage II and stage III with greater weighting of the T stage.

## Abstract 2

Supplemental Digital Content is available in the text

## INTRODUCTION

The tumor, node, metastases (TNM) staging system established by the American Joint Committee on Cancer is widely used to predict the prognosis for patients with colorectal cancer, to guide adjuvant therapy after potentially curative surgery, and to classify patients for participation in clinical trials. The ideal prognostic system should provide homogeneity within the same stage, good discrimination between different stages, and monotonicity of gradients that predicts survival outcomes that are consistent with the severity of cancer staging. Currently, the 7th edition of the TNM staging system is used in America and China,^1–3^ whereas some European countries continue to use the 5th edition.^4–6^ However, all of the TNM staging systems share similar anatomical elements and the same key principles that were inherited from the Dukes staging system that was developed in the 1932.^7^ The key principle is that patients with lymph node involvement are classified as C in the Dukes staging system and as stage III in the 7th edition TNM staging system. Thus, the principles for staging patients with colorectal cancer have been substantially stable for >80 years. It is well recognized that the Dukes B and the 7th edition TNM staging system stage II are composites of better (T3N0M0) and worse (T4N0M0) prognostic groups. Moreover, stage IIIa patients often have a better prognosis than some in stage II.^8^ In summary, the monotonicity of gradients of the existing TNM staging system for colorectal cancer is unsatisfactory.

The causes of the defect in gradient monotonicity are not well understood so far. We have reanalyzed the summary survival data of the patients from Surveillance, Epidemiology, and End Results (SEER).^3^ We rearranged all of the TN categories according to the observed survival, and then used cluster analysis to revise the staging system. According to the cluster analysis of the TN scores, T1N1a was classified as stage I, and T2N1 and T1N1b-2a were classified as stage II for both colon and rectal cancer. However, T4bN0 was classified as IIIa in colon cancer, but as IIIb in rectal cancer (Table 1). In the revised staging system, which we named the “T-plus staging system,” extra emphasis was placed on the weighting of the T stage. The SEER survival data had good monotonicity of gradients when fitted to the T-plus staging system.^3^

**TABLE 1:**
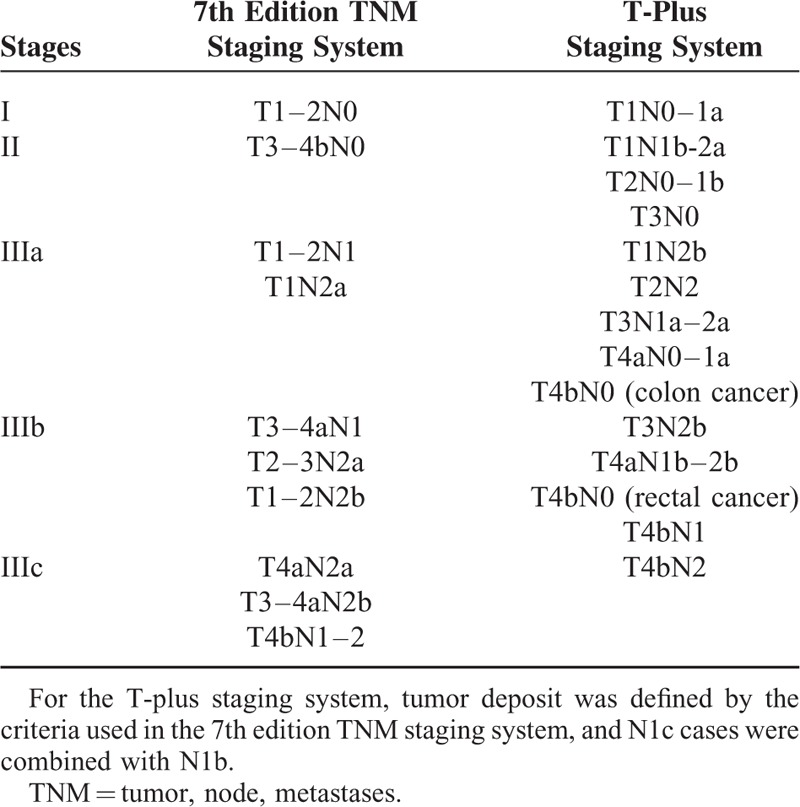
TN Categories of the 7th Edition TNM Staging System and the T-Plus Staging System for Colorectal Cancer

However, the T-plus staging system is just a proposal, and it has not been verified by individual survival data. In this study, we tested the applicability of the T-plus staging system using the data of 1 major colorectal cancer center in China.

## PATIENTS AND METHODS

### Patients

This observational study was discussed and approved by the Ethics Committee of Second Affiliated Hospital, Zhejiang University School of Medicine (SAHZU). Written informed consent was not obtained from patients in this study because we anonymized and de-identified the patients’ information prior to analysis. The colorectal carcinoma follow-up database was systematically reviewed at the Zhejiang University Cancer Institute in SAHZU. Patients who underwent colorectal carcinoma surgery from January 1985 to December 2011 were enrolled for analysis. Inclusion criteria included detailed and sufficient pathological T- and N-stage information. Exclusion criteria were death by surgical complications within a 3-month postoperative period, stage 0 or stage IV disease, multiple colorectal cancer, or prior history of malignancy. The following data were extracted: patients’ demographic and cancer pathological characteristics, surgery, perioperative complications, follow-up time and survival time.

Insofar as the T-plus staging system did not revise the stage of in situ colorectal carcinoma nor of metastatic colorectal cancer, we excluded stages 0 and IV from the present study. Tumor deposits are poor prognostic markers, and there is still obvious controversy about how to classify them. In this study, tumor deposits were defined by the criteria used in the 7th edition TNM staging system, and N1c cases were combined with N1b. The final stages were unaffected by this conversion in the 7th edition TNM staging system. Although the period of this study covered >25 years, the chemotherapy and radiotherapy protocols have changed tremendously in the past 2 decades. As a result, we omitted the adjuvant treatment and treatment for metastasis after recurrence.

### Statistical Analysis

The distributions of the measurement data were tested by skewness and kurtosis normality tests. The Gaussian distribution of the data was described by *x*^2^ ± s. Data that was not normally distributed were described by the median and inter-quartile range (M, IQR). The T stages and N stages were reviewed; then the 7th edition TNM stages and T-plus stages were calculated according to the TN categorization. Because the T-plus staging system did not subgroup stage I or stage II, this study compared stages I, II, IIIa, IIIb, and IIIc of both staging systems to keep the number of groups same.

Overall survival (OS) and disease-specific survival (DSS) were used in the survival analysis to compare the 7th edition TNM staging system with the T-plus staging system. OS was used as the primary criterion to evaluate the merits of the staging systems. OS was defined as the time from the date of the initial diagnosis to either the date of death from any cause or the date of the last follow-up. Disease-specific survival was determined as the time to death caused by colorectal cancer directly or the date of the last follow-up. Cox regression analysis was used to analyze the risks of multiple clinicopathological factors related to colorectal cancer prognosis. The stability of the Cox models in this study was tested by bootstrapping with 1000 repeats.

There was no single criterion to assess the performance of the prognostic system. The assessment was based on a comprehensive estimation that included (1) homogeneity within the same stage, that is, small differences in survival among patients in the same stage; (2) discriminatory ability between different stages, that is, greater differences in survival among patients in different stages; and (3) monotonicity of gradients, that is, the survival of patients in earlier stages for longer times than the survival of patients in more advanced stages.^9,10^

The likelihood ratio (LR) *x*^2^ test was related to the Cox proportional hazard model and used to measure homogeneity. Staging systems with higher chi-square values are the better than those with lower chi-square values. Hazard ratios (HR) and 95% confidence intervals (95% CI) were generated. The Akaike information criteria (AIC) value was calculated for each staging system to measure its discriminatory ability. A smaller AIC value indicated a better staging system. The concordance index (Harrell's c-index) was calculated to measure the capacity of the different staging systems to discriminate patients with different outcomes. The higher the c-index, the more informative the model was about a patient's outcome. Moreover, survival curves were plotted using the Kaplan–Meier method, and the log-rank test and trend *x*^2^ test were used to determine significance and to compare the discriminatory and gradient monotonicity. A higher *x*^2^ score indicated a better staging system.

All calculations were performed using the Stata 12.0 software (StataCorp LP, College Station, TX). A 2-sided *P*-value of 0.05 or less was considered to indicate statistical significance. The number of decimal places for OS, DSS, HR, AIC, and Harrell's c-index were set to 4. The number of decimal places for other characteristics was set to 2.

## RESULTS

According to the inclusion criteria, 2483 patients were reviewed. After the application of the exclusion criteria, only 2080 patients were analyzed. The median follow-up time was 60 months (IQR 40–85 months). The baseline characteristics of patients in this study were listed in Table 2. A total of 827 patients received adjuvant therapy, including chemotherapy and/or radiotherapy. Among them, 78 rectal cancer patients received adjuvant radiotherapy. For 2080 colorectal cancer patients, Cox regression analysis of multiple clinicopathological parameters against overall survival showed that independent risk factors included rectal cancer, older ages, non-R0 resection, mucinous adenocarcinoma or signet-ring cell carcinoma, fewer harvested lymph nodes, higher T stages, higher N stages, and higher preoperative carcinoembryonic antigen (CEA) level (Supplementary Table 1). The patients were staged respectively by the 7th edition TNM staging system and the T-plus staging system. With the 7th edition TNM staging system, the number of patients in stage IIIa was the smallest. With the T-plus staging system, the number of patients in stage I and IIIc was the smallest. With the 7th edition TNM staging system, the 5-year OS of patients in stage IIIa (0.8615, Table 2) indicated a better prognosis than for patients in stage II (0.7531, Table 2). Similarly, the 7th edition TNM staging system also indicated a better prognosis for the 5-year DSS for patients in stage IIIa (0.8615, Table 2) than for patients in stage II (0.7729, Table 2). This indicated that the 7th edition TNM staging system was faulty in generating a monotonic gradient for the prediction of OSS and DSS. In contrast, the 5-year OSS and DSS predicted by the T-plus staging system declined monotonically as the stages increased from I to IIIc (Table 2).

**TABLE 2:**
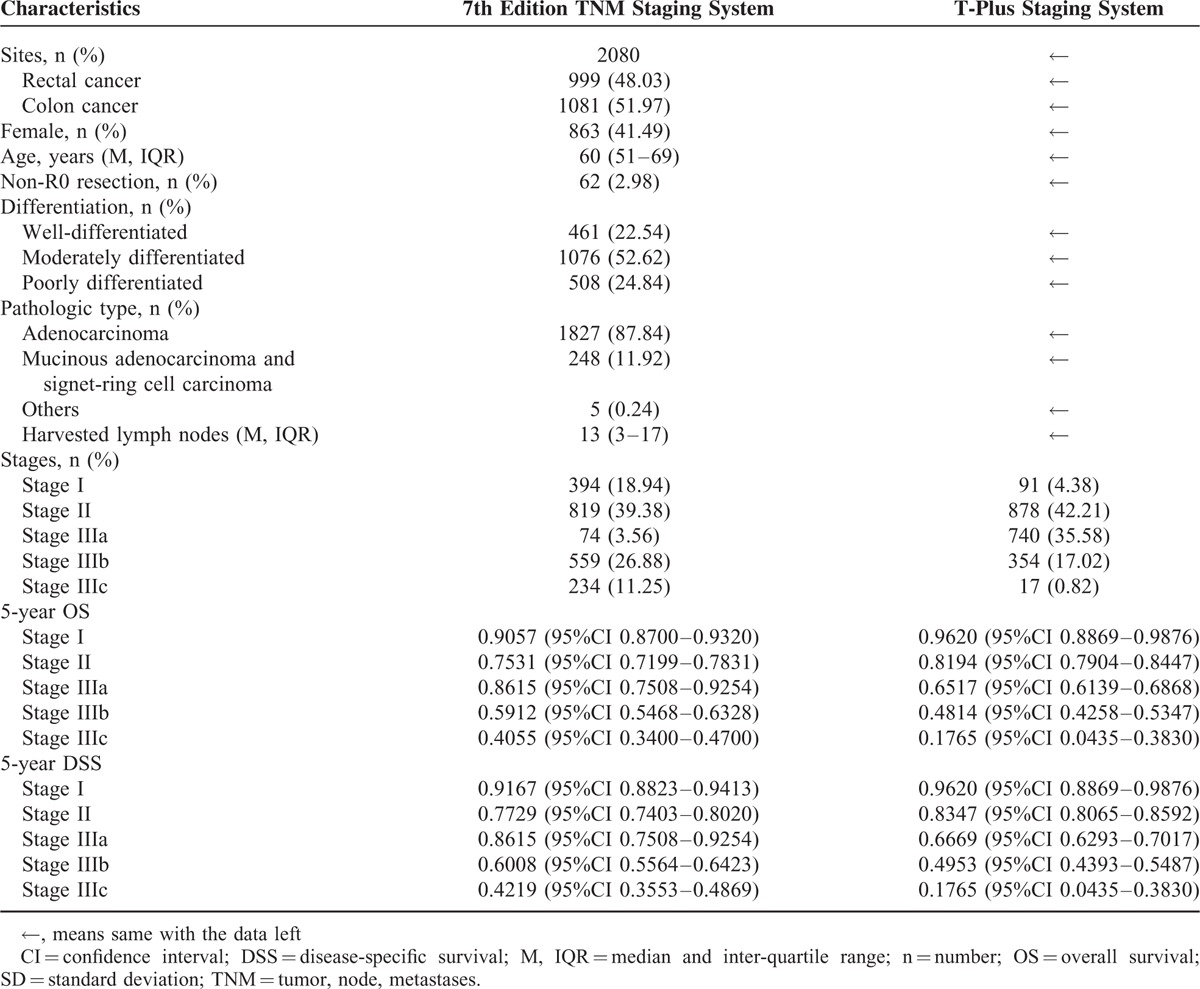
Baseline Demographic and Cancer Characteristics of the Patients

**FIGURE 1:**
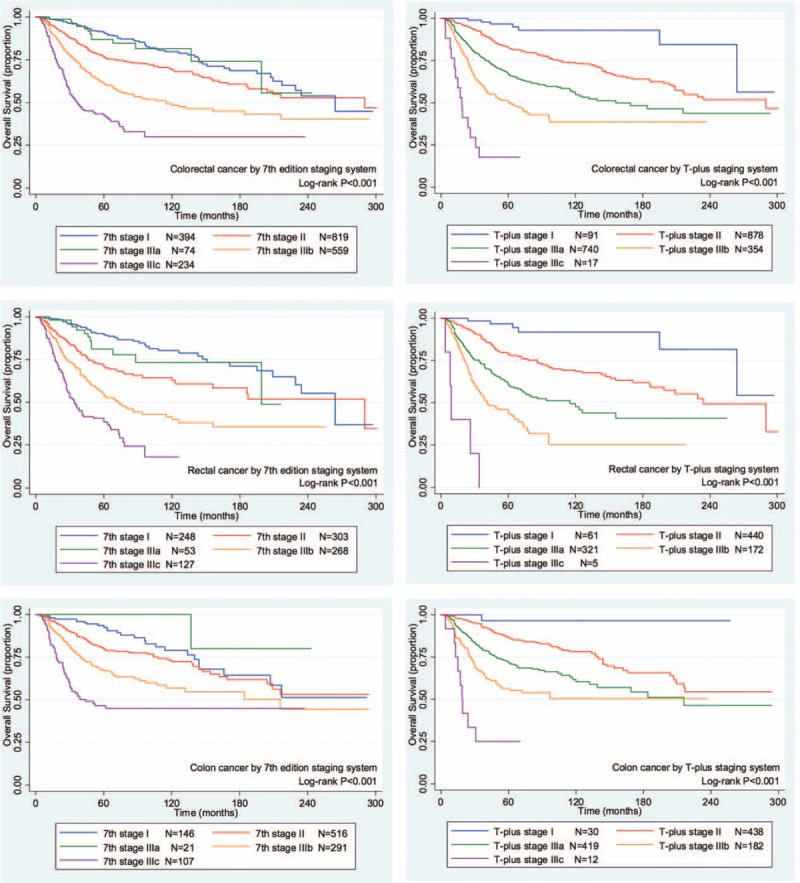
The Kaplan–Meier overall survival curves of colorectal cancer patients for the 2 staging systems. Left column, 7th edition TNM staging system; right column, T-plus staging system; first row, colorectal cancer; second row, rectal cancer; third row, colon cancer.TNM = tumor, node, metastases.

**FIGURE 2:**
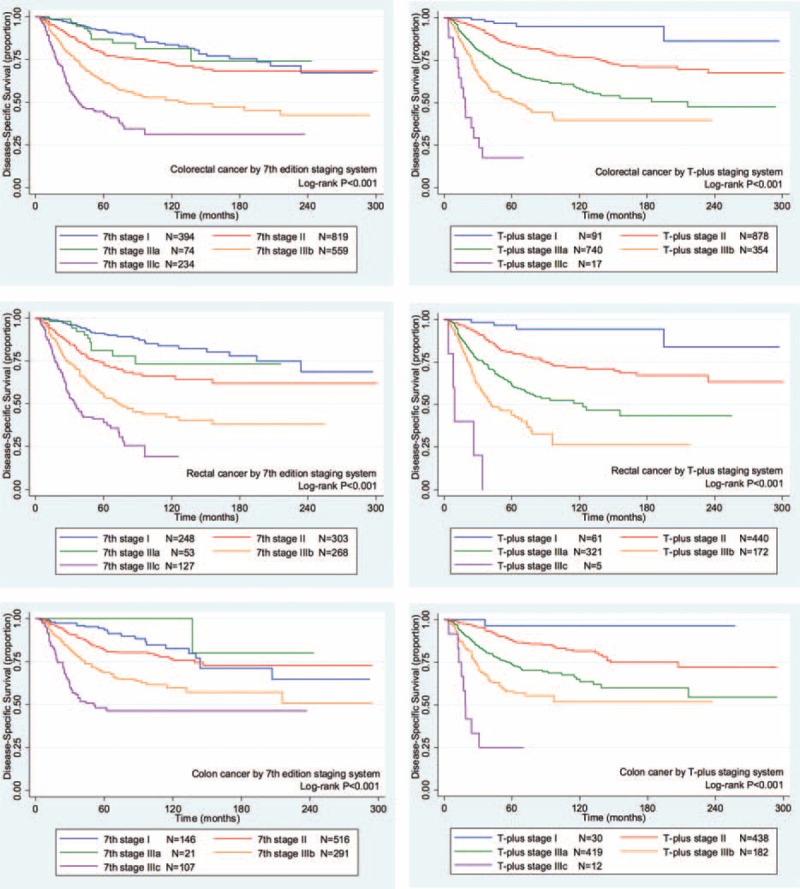
The Kaplan–Meier disease-specific survival curves of colorectal cancer patients for the 2 staging systems. Left column, 7th edition TNM staging system; right column, T-plus staging system; first row, colorectal cancer; second row, rectal cancer; third row, colon cancer.TNM = tumor, node, metastases.

For all of the colorectal cancer patients, the 2 staging systems showed similar homogeneity and discriminatory ability with a slight advantage in the T-plus staging system. The T-plus staging system predicted an OS HR (1.9663) that was higher than that predicted by the 7th edition TNM staging system (1.5120, Table 3). Similarly, the HR for the DSS calculated by the T-plus staging system was higher than that for the 7th edition TNM staging system (Table 4). To more closely examine the monotonicity of the survival curves for the different stages, we constructed OS and DSS Kaplan–Meier curves. For the T-plus staging system, stage IIIa and stage II were clearly differentiated for both OS (Figure 1) and DSS (Figure 2), and the patients with higher stages showed poorer prognoses. For the T-plus staging system, none of the survival curves for any of the stages crossed. However, for the 7th edition TNM staging system, the OS (Figure 1) and DSS (Figure 2) curves of stage IIIa crossed with stage I.

**TABLE 3:**
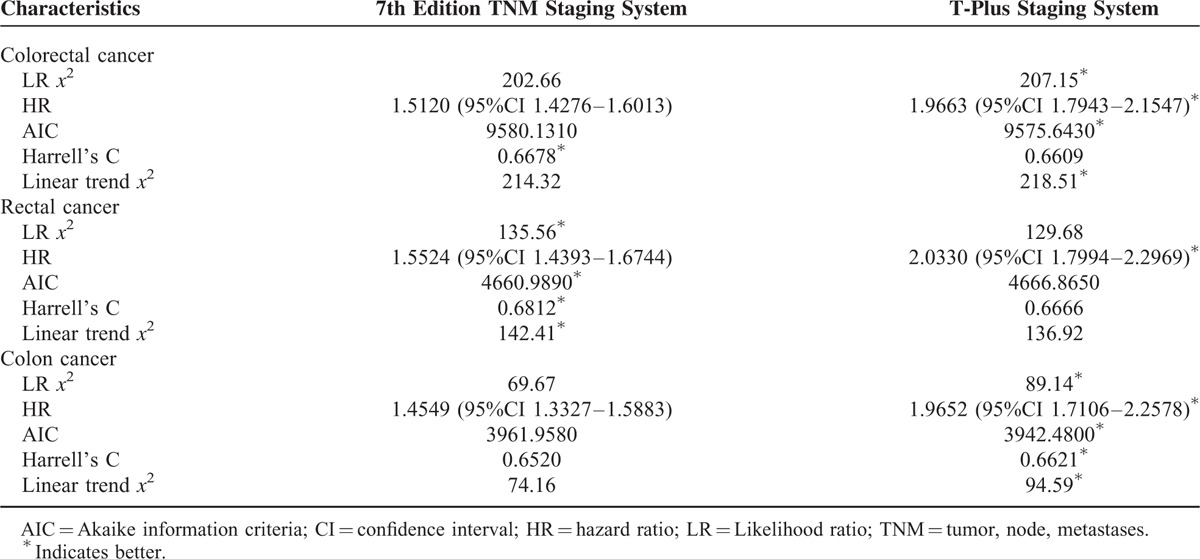
Comparison of the Performance of 2 Staging System by Overall Survival

**TABLE 4:**
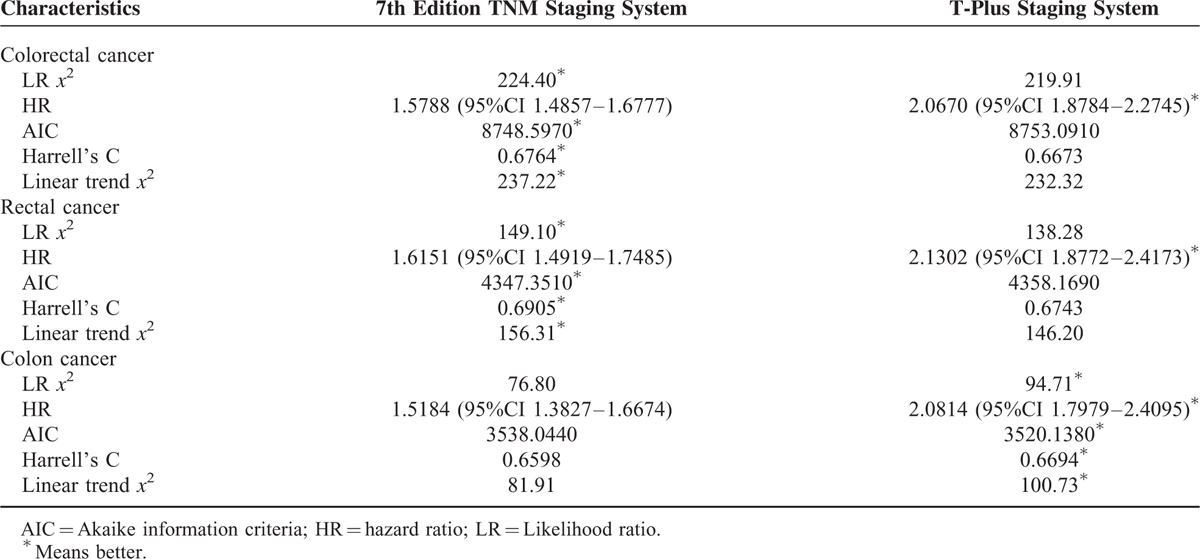
Comparison of the Performance of 2 Staging System by Disease-Specific Survival

For patients with rectal cancer, the homogeneity and discriminatory ability of the T-plus staging system was similar with, but not quite as good as, the 7th edition TNM staging system. However, the OS HR predicted by the T-plus staging system, 2.0330 (Table 3), was higher than that predicted by the 7th edition TNM staging system, 1.5524. Likewise, the DSS HR predicted by the T-plus staging system, 2.1302 (Table 4), was higher than that predicted by the 7th edition TNM staging system, 1.6151. For the OS gradient monotonicity (Figure 1) and DSS gradient monotonicity (Figure 2) determined by the 7th edition TNM staging system, the Kaplan–Meier curve for stage IIIa lay between the curves for stage I and stage II. However for the T-plus staging system, the gradient monotonicity of both the OS and DSS was well-associated with the severity of the stages.

For patients with colon cancer, the homogeneity and discriminatory ability of the T-plus staging system was better than that for the 7th edition TNM staging system. The OS HR predicted by the T-plus staging system, 1.9652 (Table 3), was higher than that predicted by the 7th edition TNM staging system, 1.4549. Likewise, the DSS HR predicted by the T-plus staging system, 2.0814 (Table 4), was higher than that predicted by the 7th edition TNM staging system, 1.5184. For the gradient monotonicity of the OS (Figure 1) and DSS (Figure 2) predicted by the 7th edition TNM staging system, the Kaplan–Meier curve for stage IIIa lay beyond the curve of stage I. However, for the T-plus staging system, the gradient monotonicity was consistent with the severity of the stages.

## DISCUSSION

There is a survival paradox between stage IIIa and stage II in the 7th edition TNM staging system for colorectal cancer. The phenomenon has been observed in SEER, Japanese, and Korean studies, as well as our own.^1,2,3,11,12^ These data point to an inherent defect of the existing TNM staging system in which there is poor monotonicity of gradients from the early stages to the advanced stages. The reason for this peculiarity is not known with certainty. We proposed that the defect lies in the overestimated weighting of the N stage. Consequently, we proposed a revision of the TNM staging system, called the T-plus staging system.^3^ Our proposed system assigned more weight to the T stage than did the 7th edition TNM staging system, which itself in 2010 increased the weighting of the T stage over the 6th edition TNM staging system. Colorectal cancer, which directly infiltrates or is adherent to organs or structures, is associated with worse prognoses. For this reason, stage T4a and T4b were added to stratify the stage T4 in the 7th edition TNM staging system. T4bN0 lesions were redefined from stage IIb to IIc. Similarly, T1–2N2 was reclassified from IIIc to IIIa/IIIb.^8^ These modifications reflect that the weight of T stage in the TNM staging system increasingly enhanced in colorectal cancer patients than previously believed. This has also been confirmed by the data from the United Kingdom, Korea, and Iceland. ^13–15^ Roxburgh et al found that only T stage, no other pathology features, was associated with survival time when venous invasion absent in stage I to III colorectal cancer. In addition, the combination of T stage and venous invasion had similar predictive value as the TNM staging system for node-positive tumors and superior predictive value than the TNM staging system for node-negative tumors.^13^ Kim et al reported that the pT4 status was the most significant pathologic determinant of poor outcome, comparing to the other 25 histopathologic and immunohistochemical factors, in patients with stage II/III microsatellite instability–high colorectal cancer regardless of the regimens of adjuvant chemotherapy.^14^ The Iceland nationwide retrospective study also found that pT4 was a major indicator of poor prognosis in stage II/III colon carcinoma. Moreover, they emphasized that 4-tiered TNM or Dukes staging systems were insufficient by not taking this variable into account.^15^ As the emphasis by Iceland study, the key principle that defines stage III in existing TNM staging system according to the status of the lymph nodes has never been changed since 1932. The T-plus staging system abolishes this principle and makes the T stage more important. For example, T1N1a is classified as stage I, and T2N1 and T1N1b-2a are classified as stage II for both colon and rectal cancer. However, T4bN0 is classified as IIIa in colon cancer, but as IIIb in rectal cancer.

So far, the T-plus staging system has just been debated without being verified. SAHZU is one of the major colorectal cancer centers in China and the lead institution of the Committee of Colorectal Cancer of Chinese Anti-Cancer Association. The database of colorectal cancer in SAHZU has >25 years of follow-up data. This we used the patient data in this resource to test the applicability of the T-plus staging system.

Comprehensive assessment of the performance of the staging systems included (1) homogeneity within the same stage, (2) discriminatory ability between different stages, and (3) monotonicity of gradients. For colorectal cancer, performance of the T-plus staging system was similar to the 7th edition TNM staging system with respect to homogeneity within the same stage and discriminatory ability between different stages. However, for colon cancer, the T-plus staging system was clearly better.

For gradient monotonicity, the T-plus staging system was superior to the 7th edition TNM staging system for both colon and rectal cancer. According to the Kaplan–Meier survival curves, the T-plus staging system discriminated patients to different stages and the corresponding survival decreased following the increased severity of the stages. However, for the 7th edition TNM staging system, stage IIIa showed better a prognosis than stage II for rectal cancer and better than stage I for colon cancer. In summary, the T-plus staging system showed similar homogeneity and discriminatory ability but better monotonicity of gradients than the 7th edition TNM staging system for colorectal cancer.

In the past 3 decades, the adjuvant chemotherapy and radiotherapy have progressed significantly which improved the survival of colorectal cancer patients. It is naturally to be argued that the systemic treatment improved the survival of stage IIIa patients which result the paradox between stage IIIa and stage II in the 7th edition TNM staging system. In this study, we did not stratify the patients by systemic treatment considering that the regimen changed tremendously in the past time and the palliative treatment also affects the overall survival which made the stratification difficult and unreliable. However, we have analyzed the performance of both staging systems for the patients diagnosed before 2005 (total 1100 patients) and after 2005 (total 980 patients) separately considering Oxaliplatin has been used widely since then. The not-shown results were consisted with the results above showed which indicated that the paradox was the inherent defect of the 7th edition TNM staging system instead of the affect of the systemic treatment. Moreover, the stage II and III rectal cancer, which were classified as locally advanced rectal cancer, received the same adjuvant radiochemotherapy and the stage IIIa patients still showed better prognosis than patients stage II.^3^ This also testify that the paradox was derived from the 7th edition TNM staging system not adjuvant therapy.

So far, the T-plus staging system has been verified to work well. However, it is not a flawless system for 3 reasons. First, currently the tumor deposit is a poor prognostic marker, and the weighting of it should be re-evaluated in a future staging system. However, there are obvious concerns about how to classify a tumor deposit. In the T-plus staging system, we did not propose any new criteria to define the tumor deposit, but rather we followed the definition used in the 7th edition TNM staging system. Tumor deposits first emerged as prognostic indicators in the 5th edition TNM staging system in 1997. In that system, the 3-mm rule was used to define the tumor deposit. For the 6th edition TNM system published in 2002, the contour criteria replaced the 3-mm rule to define the tumor deposit. In the 7th edition TNM staging system published in 2010, the definition of the tumor deposit depends on the diagnosis of the pathologist instead of high level evidence. Thus, it has changed over recent editions of the TNM staging systems. Recently, it has been reported that the 5th edition TNM staging system is the best fit to define such tumor deposits in colorectal cancer.^4–6,9,16^

The second flaw in the T-plus staging system is that the distribution of patients in different stages is not equal. The number of patients of stage I and stage IIIc is obviously less than that in stage II and stage IIIa/b. The problem is more obvious for rectal cancer. This may be the reason that the T-plus staging system did not show any advantage in discriminatory ability (reflected by AIC) for rectal cancer in spite of the fact that the Kaplan–Meier survival curves discriminate among the different stages perfectly. There are 2 possible causes to the problem: (1) screening for early stage colorectal cancer was not legally required by the medical insurance in China. As a result, stage I (T1N0–1a) patients were rarely identified, and (2) stage IIIc was defined as only T4bN2, and only a few such patients had involved adjacent organs. The small number of stage I patients made it impossible to define and identify subgroups within that state.

The third flaw in the T-plus staging system is that it does not define and identify any subgroups within stage II.^3^ Thus, there remains a need to determine whether or not subgroups in stage II are necessary and how to identify them. Moreover, this study was based on a retrospective study of the database of a single center. As a result, the surgical quality, adjuvant therapy, and salvage chemotherapy were not controlled prospectively. Prior to 2010, the proportion of rectal cancer patients in China who received radiotherapy was lower than that in western countries.^17,18^ The median age of the patients in our database was younger than the population of similar patients of western countries. Thus, the results of this study might have been affected by biases within the database. Consequently, the reliability of the T-plus staging system for colorectal cancer should be verified by prospective studies and the databases of more centers.

The existing TNM staging/Dukes staging system was widely used to predict the prognosis of colorectal cancer because it was feasible and simple to use for physicians all of the world. However, the existing TNM staging system is not perfect and has not kept up with the times considering that it has been stable since the 1930s. That system considers only 2 anatomic factors: invasion depth and lymph node metastasis. In recent decades, more pathological factors, for example, venous or lymphatic invasion and perineural invasion; cellular factors, for example, poor differentiation, mucinous adenocarcinoma or signet-ring cell carcinoma, and high CEA level; and molecular factors, for example, ras gene mutation, B-raf gene mutation, microsatellite instability, and CpG island methylator phenotype, have been confirmed to be associated with the prognosis of colorectal cancer.^19,20^ However, the existing TNM staging system does not take the above factors into consideration. The key problem is how to comprehensively analyze and optimally analyze such complex prognostic factors. Our team is working to try to establish a nonlinear prognosis predictive model that integrates more prognosis-related factors to better predict the survival of colorectal cancer patients. We hope to provide professionals with this model as a mobile app in the future to make the calculations easy to access.

This is the first direct evidence to support the need to abolish the discrimination of stages II and III in colorectal cancer based on lymph node status. The T-plus staging system is similar to the concept used in the TNM staging system for gastric cancer in which N1 also can be classified into stage I.^8^ The creativity of the T-plus system is to classify patients with colorectal cancer by overall survival instead of by artificial criteria as done by the Dukes staging system. The T-plus staging system is not perfect, but it works very well, especially with gradient monotonicity. We propose that the T stage should be given more weighting in any future colorectal cancer staging system.

## Supplementary Material

Supplemental Digital Content
