# Supplementary material for: TNM Staging of Colorectal Cancer Should be Reconsidered According to Weighting of the T Stage: Verification Based on a 25-Year Follow-Up
Source: Medicine (Baltimore). 2016 Feb 12;95(6):e2711. doi: 10.1097/MD.0000000000002711 (PMC4753906; doi:10.1097/MD.0000000000002711)
Supplement: Supplemental Digital Content [file medi-95-e2711-s001.doc]

**Supplementary Table 1 Cox regression analysis of multiple clinicopathological parameters of 2080 colorectal cancer patients**

| **Characteristics** | **HR** | **Bootstrap SE** | **Z** | ***P*>|Z|** | **95%CI** |
| --- | --- | --- | --- | --- | --- |
| **Sites**  (Rectal *vs.* colon) | 1.4400* | 0.1378 | 3.81 | <0.001 | 1.1938-1.7370 |
| **Age**  (older *vs.* younger) | 1.0158* | 0.0045 | 3.56 | <0.001 | 1.0071-1.0246 |
| **Gender**  (male *vs.* female) | 1.1531 | 0.1088 | 1.51 | 0.131 | 0.9584-1.3873 |
| **R0 resection**  (yes *vs.* no) | 0.3195* | 0.0799 | -4.56 | <0.001 | 0.1956-0.5216 |
| **Differentiation grades** | 0.9992 | 0.0839 | -0.01 | 0.993 | 0.8475-1.1781 |
| **Histological types**  (mucinous adenocarcinoma or signet-ring cell carcinoma *vs.* adenocarcinoma) | 1.3640* | 0.2081 | 2.03 | 0.042 | 1.0114-1.8395 |
| **Venous or lymphatic invasion**  (yes *vs.* no) | 1.0643 | 0.2034 | 0.33 | 0.744 | 0.7318-1.5478 |
| **Perineural invasion**  (yes *vs.* on) | 0.8842 | 0.2409 | -0.45 | 0.651 | 0.5183-1.5082 |
| **Harvested lymph nodes** | 0.9643* | 0.0074 | -4.76 | <0.001 | 0.9499-0.9788 |
| **T stages** | 1.5135* | 0.0854 | 7.35 | <0.001 | 1.3552-1.6904 |
| **N stages** | 1.4237* | 0.0543 | 9.26 | <0.001 | 1.3211-1.5342 |
| **Preoperative CEA level** | 1.0032* | 0.0014 | 2.25 | 0.024 | 1.0004-1.0060 |
| **Adjuvant therapy** | 0.9631 | 0.1015 | -0.36 | 0.721 | 0.7833-1.1841 |

* Indicates statistical significance; HR, hazard ratio; SE, standard error; CI, confidence interval; CEA, carcinoembryonic antigen.
